# The Upregulation of L1CAM by SVHRSP Mitigates Neuron Damage, Spontaneous Seizures, and Cognitive Dysfunction in a Kainic Acid-Induced Rat Model of Epilepsy

**DOI:** 10.3390/biom15071032

**Published:** 2025-07-17

**Authors:** Zhen Li, Biying Ge, Haoqi Li, Chunyao Huang, Yunhan Ji, Melitta Schachner, Shengming Yin, Sheng Li, Jie Zhao

**Affiliations:** 1 National-Local Joint Engineering Research Center for Drug Research and Development (R&D) of Neurodegenerative Diseases, Dalian Medical University, Dalian 116044, China; 2 Functional Laboratory, College of Basic Medical Sciences, Dalian Medical University, Dalian 116044, China; 3 Department of Cell Biology and Neuroscience, Keck Center for Collaborative Neuroscience, Rutgers, The State University of New Jersey, Piscataway, NJ 08854, USA; 4 Liaoning Provincial Key Laboratory of Cerebral Diseases, Department of Physiology, College of Basic Medical Sciences, Dalian Medical University, Dalian 116044, China; 5 Department of Biochemistry, School of Basic Medical Science, Dalian Medical University, Dalian 116044, China

**Keywords:** scorpion venom, peptide, SVHRSP, TLE, L1CAM, cell adhesion molecule, neuroprotection

## Abstract

Temporal lobe epilepsy (TLE) is a common drug-resistant form of epilepsy, often accompanied by cognitive and emotional disturbances, highlighting the urgent need for novel therapies. Scorpion Venom Heat-Resistant Synthetic Peptide (SVHRSP), isolated and synthetically derived from scorpion venom, has shown anti-epileptic and neuroprotective potential. This study evaluated the anti-epileptic effects of SVHRSP in a kainic acid (KA)-induced TLE rat model. Our results demonstrated that SVHRSP (0.81 mg/kg/day) reduced the frequency and severity of spontaneous seizures. Behavioral tests showed improved cognitive performance in the novel object recognition, object location, and T-maze tasks, as well as reduced anxiety-like behavior in the open-field test. Moreover, SVHRSP mitigated hippocampal neuronal loss and glial activation. Transcriptomic analysis indicated that SVHRSP upregulates genes involved in adhesion molecule-triggered and axon guidance pathways. Western blotting and immunofluorescence further confirmed that SVHRSP restored dendritic (MAP2), axonal (NFL), and synaptic (PSD95) marker expression, elevated the functionally important L1CAM fragment (L1-70), and increased myelin basic protein-induced serine protease activity responsible for L1-70 generation. Blockade of L1CAM expression diminished the neuroprotective effects of SVHRSP, suggesting a critical role for L1CAM-mediated synapse functions. This study is the first to reveal the therapeutic potential of SVHRSP in TLE via L1CAM-associated mechanisms.

## 1. Introduction

Epilepsy is a chronic neurological disorder characterized by abnormal neuronal discharges and excessive network synchronization, with approximately one-third of patients being resistant to current anti-epileptic drugs (AEDs) [1]. Poor seizure control not only increases seizure frequency and severity but is also frequently accompanied by cognitive impairment, psychiatric disorders, and epilepsy-related sudden death, severely compromising patients’ quality of life. Temporal lobe epilepsy (TLE) is the most common form of drug-resistant epilepsy, often associated with hippocampal sclerosis [2], which is characterized by selective neuronal loss in the hippocampal CA1–CA4 regions and dentate hilus, along with gliosis [3,4]. Recent studies suggest that neuronal dysfunction, synaptic connectivity abnormalities, and neural network remodeling play critical roles in the pathogenesis and progression of TLE [5]. In addition, brain injuries such as traumatic brain injury, ischemic stroke, or status epilepticus often result in extensive neuronal loss, which not only predisposes those affected to epilepsy but also contributes to impaired learning, memory deficits, and cognitive decline [6]. Current AEDs primarily act by inhibiting abnormal neuronal activity but have limited effects on disease pathology [7]. Thus, the development of novel therapeutic strategies that can simultaneously suppress seizures and ameliorate neuronal dysfunction as well as cognitive impairment remains a major challenge [8].

Neurites, including axons and dendrites, are slender projections that form the structural foundation for inter-neuronal communication [9]. Axons are responsible for transmitting action potentials over long distances to target cells, while dendrites receive synaptic inputs from other neurons. Synapses are specialized structures that mediate chemical or electrical communication between neurons, and their proper formation and localization are essential for the functional integrity of neural circuits. The assembly of synapses relies not only on the physical contact between neurites but also on complex molecular recognition and signaling processes [10]. Cell adhesion molecules (CAMs) play crucial roles in the precise assembly and molecular organization of synapses. CAMs can form synaptic contacts through trans-synaptic interactions and regulate synaptic plasticity. These molecules are not only essential for maintaining normal synaptic structure and function but also control the functionality of synaptic receptors and participate in synapse elimination [11].

L1 cell adhesion molecule (L1CAM), a member of the immunoglobulin superfamily, is a transmembrane protein composed of an extracellular N-terminal region, a single transmembrane domain, and a short intracellular C-terminal segment [12,13]. L1CAM is involved in the development of the nervous system [14] by regulating neuronal proliferation, migration, and differentiation [12], axon and dendrite formation [15], synaptic stabilization [11], myelination [16], and action potential generation [17]. The myelin basic protein (MBP) cleaves L1CAM at the Arg687 site within its extracellular domain to produce the transmembrane fragment L1-70, a process essential for neurite outgrowth, neuronal survival, and migration, as evidenced by impaired L1-70 production and neurological dysfunction in MBP-deficient mice [18]. L1CAM localizes to presynaptic terminals in the hippocampus and influences the development of the central and peripheral nervous systems through its multifunctional roles. However, its involvement in epilepsy has not been studied.

Scorpion venom is a rich source of bioactive molecules, containing a variety of pharmacologically active components, most of which are peptides [19,20]. Based on their structural characteristics, scorpion venom peptides can be classified into disulfide-bridged peptides (DBPs) and non-disulfide-bridged peptides (NDBPs). DBPs possess a unique fixed 3D structure, enabling them to interact specifically with ion channels, and their potential roles in medicine have been intensively studied [21]. However, their toxicity hampers possible clinical applications. In contrast, NDBPs have a more elastic structure and cannot bind to ion channels as efficiently as DBPs [22], and hence fewer studies have focused on them. In view of these considerations, we have produced a novel peptide construct of 15-amino-acid NDBP, named Scorpion Venom Heat-Resistant Synthetic Peptide (SVHRSP), from heat-treated venom of the Chinese *Buthus martensii Karsch* scorpion. Previous studies have demonstrated that SVHRSP can mitigate epileptic [23] and Parkinson’s disease symptoms in mice [24], and that it exhibits high biosafety. To further investigate its effects on chronic epilepsy and the accompanying cognitive impairment, we studied the neuroprotective effects of SVHRSP in a kainic acid (KA)-induced chronic epilepsy model, with a particular focus on its potential association with L1CAM in the pathogenesis of epilepsy. This work may provide new targets and strategies for the treatment of epilepsy.

## 2. Material and Methods

### 2.1. Peptide Synthesis

The amino acid sequence of SVHRSP is KVLNGPEEEAAAPAE (molecular weight: 1524.7 Da), and the sequence has been patented (patent number: ZL201610645111.7). In this study, SVHRSP was synthesized by GL Biochem Ltd. (Shanghai, China) with a purity of 98%.

### 2.2. Animals

Animal studies are reported in compliance with the ARRIVE guidelines [25]. Animals were singly housed in a controlled environment (temperature set at 24–26 °C and relative humidity set at 40–70%). Male Sprague Dawley (SD) rats, 7–9 weeks old, weighing 270–320 g were from Vital River Laboratory Animal Technology Co. (Beijing, China). The animals were acclimatized to the environment for a week before the experiments. Lighting in the animal rooms was kept alternately bright and dark for 12 h, and air changes were maintained at a minimum of 10 times/hour. The animal experimental protocol was approved by the Institutional Animal Care and Use Committee (Approval No: GP02-083-2020v1.0), and the animal experimental procedures followed the “Guidelines for the Welfare and Ethics of Laboratory Animals in China”.

### 2.3. Electrode Implantation and EEG Recording

After a 7-day acclimatization period, animals underwent electrode implantation surgery. On the day of surgery, anesthesia was induced with 3% isoflurane (ORBIEPHARM, Beijing, China) in 0.5 L/min oxygen until the animal reached a deep anesthetic state, as indicated by stable respiration, muscle relaxation, and a lack of response to painful stimuli. Anesthesia was then maintained with 2% isoflurane in 0.5 L/min oxygen. The rats were positioned in a stereotaxic apparatus (71000, RWD Life Science, Shenzhen, China). Three burr holes were drilled into the skull: two recording electrodes were placed 1.5 mm lateral to the midline and 2 mm anterior to the bregma, and the reference electrode was positioned 1.5 mm lateral to the midline and 2 mm posterior to the bregma. Electrodes (diameter: 1 mm, length: 3 mm) were inserted into the burr holes and secured with dental acrylic. Following surgery, animals were allowed to recover for 7 days before EEG recordings were initiated. On the final day of recovery, 24 h baseline EEG recordings were obtained from all animals.

### 2.4. KA Model of Epilepsy

Male SD rats were intraperitoneally injected with kainic acid (KA, 10 mg/kg; K0250, Sigma, St. Louis, MO, USA) to induce epilepsy. Seizure behaviors wase monitored for 3 h post-injection and evaluated using the Racine scale [26]: category 1, immobility and facial twitch; category 2, head nodding; category 3, forelimb clonus; category 4, rearing; and category 5, rearing and falling. Only animals that developed Racine stage 4–5 seizures within 1 h of KA administration and exhibited status epilepticus (SE) lasting at least for 2 h were included in the study. Three hours after SE termination, animals were randomly assigned to receive either SVHRSP (810 μg/kg, subcutaneously, daily for 7 consecutive days) or vehicle (saline). Starting from day 7 post-induction, 12 h EEG recordings (AD Instruments, Dunedin, New Zealand) and video monitoring (HIKVISION, Hangzhou, China) were performed daily for each group to observe seizures and spontaneous recurrent seizures (SRS), which were continuously recorded. The incidence of SRS, total number of seizures, seizure duration, and seizure severity for each rat were assessed and documented by two experienced investigators blinded to the treatment groups.

### 2.5. Open-Field Test (OFT)

The OFT was used to evaluate spontaneous locomotor activity, exploratory behavior, and anxiety levels in a novel environment. The test apparatus consisted of a black-walled open-field box (80 cm × 80 cm × 40 cm) with the floor divided evenly into 16 squares as documented by others [27]. All experiments were carried out in a quiet environment. At the beginning of each trial, rats were gently grasped by the base of the tail and placed into the lower left quadrant of the center area. Immediately afterwards, the experimenter exited the testing room, and behavioral recording was initiated using an automated tracking system (RWD Life Science, Shenzhen, China), which recorded the animals’ activities for 5 min. The analyzed parameters included the total distance traveled and the time spent in the central area. After each trial, the open-field apparatus was thoroughly cleaned with 75% ethanol to eliminate odors.

### 2.6. Object Recognition Test (ORT)

The ORT was employed to assess non-spatial cognitive abilities in experimental animals. The testing apparatus consisted of a transparent open-top plastic box (80 cm × 80 cm × 40 cm), isolated from the surrounding environment with curtains and placed within a sound-attenuated room. Three objects were used: objects A and B were identical, while object C was different. The ORT training was conducted 24 h after the completion of the OFT experiment. On the first day of testing, the rats were allowed to explore objects A and B for 5 min. Twenty-four hours after the training session, one of the familiar objects was replaced with object C, and the rats were allowed to explore for 5 min during the test session, which was video-recorded. Between each trial, the box and objects were cleaned with 75% ethanol to eliminate olfactory cues. Upon completion of all behavioral assessments, rats were returned individually to their home cages. Non-spatial cognitive performance was measured using the discrimination index (DI), calculated as DI = (Tn − Tf)/(Tn + Tf), where Tn and Tf represent the time spent exploring the novel and familiar objects, respectively.

### 2.7. T-Maze Spontaneous Alternation Test

The T-maze offers clear advantages in detecting hippocampus-dependent working memory deficits [28]. In addition, its design allows for precise control of the starting arm, enabling consistent task standardization and minimizing variability between trials. Therefore, 24 h after the completion of the ORT, we selected the T-maze to assess working memory and spontaneous alternation behavior, as reported in previous studies [29]. The T-maze apparatus consisted of three arms made of black opaque material: one start arm and two goal arms, each measuring 40 cm in length, 10 cm in width, and 20 cm in height. Prior to testing, rats were habituated to the apparatus for 10 min. During the test session, each rat was placed at the end of the start arm and allowed to freely explore the maze. An alternation was defined as the rat entering one goal arm first, and then entering the opposite goal arm on the next trial. In other words, a successful alternation required the rat to visit the arm opposite to the one it had previously entered. Each test session consisted of 8 trials per animal, with each trial lasting until the animal made a choice. Each trial usually lasted for 60 s. If a trial exceeded this time without a choice, the animal was not included in the test. The sequence of arm entries was manually recorded. The spontaneous alternation percentage was calculated using the following formula: Alternation (%) = (number of alternations/[total number of arm entries − 2]) × 100%. Between tests, the maze was thoroughly cleaned with 75% ethanol to eliminate olfactory cues.

### 2.8. Object Location Test (OLT)

The OLT was performed to evaluate spatial memory. The testing apparatus and environmental conditions were identical to those used in the ORT. Two identical objects were used in this experiment, being distinct from those used in the object recognition task. On the first day, rats were habituated to the apparatus without objects. On the following day, two identical objects were placed in fixed positions within the box, and the rats were allowed to explore them freely for 5 min. After a 24 h retention interval, one of the objects was relocated to a novel position, while the other remained in its original location. Rats were then subjected to a 5 min exploration test session, which was video-recorded. Between each trial, the apparatus and objects were thoroughly cleaned with 75% ethanol to eliminate olfactory traces. Spatial memory was quantified using the DI, calculated as DI = (Tn − Tf)/(Tn + Tf), where Tn represents the time spent exploring the relocated (novel) object and Tf represents the time spent exploring the stationary (familiar) object.

### 2.9. RNA Sequencing, Differential Gene Analysis, and Pathway Enrichment

For transcriptomic analysis, animals were sacrificed on day 14 after KA induction. They were euthanized by rapid cervical dislocation to ensure immediate death. Total RNA was extracted from hippocampal and cortical tissues using TRIzol reagent (AG21101, Accurate Biology, Changsha, China) according to the manufacturer’s instructions. RNA concentration and purity were determined by spectrophotometry, and all samples exhibited a 260/280 nm absorbance ratio between 1.9 and 2.0. RNA samples from the hippocampus and cortex of three rats per group were collected and sent to Novogene Co., Ltd. (Beijing, China) for sequencing. After an initial quality control (RNA concentration and purity were assessed by Nanodrop spectrophotometry, with 260/280 nm ratios between 1.8 and 2.0), RNA integrity was further evaluated using the Agilent Bioanalyzer Nano-ChIP system (Agilent Technologies, Santa Clara, CA, USA), and samples with an RNA Integrity Number (RIN) ≥ 9 were selected for library preparation. Messenger RNA was enriched using oligo (dT) magnetic beads. Libraries were constructed and samples were quantified using an adapter-specific qPCR kit, and equimolar amounts of each library were pooled and sequenced on an Illumina HiSeq 2000 platform. Gene-expression levels were estimated as fragments per kilobase of transcript per million mapped reads (FPKM). Differential gene-expression analysis was performed using the DESeq2 package (version 1.36.0) in the R environment (version 4.4.3). Genes with a *p*-value < 0.05 and an absolute log2 fold change >1 were considered significantly differently expressed. Gene Ontology (GO) and Kyoto Encyclopedia of Genes and Genomes (KEGG) pathway enrichment analyses were conducted using the DAVID database (https://david.ncifcrf.gov/, accessed on 18 March 2025)) based on the rat genome annotation. Gene Set Enrichment Analysis (GSEA) was performed using ranked gene lists based on log-fold changes.

### 2.10. Immunohistochemistry

Rats in each group were terminally anesthetized with 1% sodium pentobarbital (6 mL/kg); the brains were perfused transcardially with pre-cooled saline and 4% formaldehyde. The brains were post-fixed with 4% formaldehyde at 4 °C for 48 h and then transferred sequentially into 10%, 20%, and 30% sucrose solutions. Embeddings in each sucrose step clasted 12 h until the tissue sank to the bottom of the container and then frozen for cryo-sectioning. Each brain was coronally cut into 30-μm-thick slices based on the rat brain atlas (hippocampus: interaural 6.00–5.52 mm, Bregma-3−3.48 mm), using a cryostat (Leica Microsystems, Wetzlar, Germany). Sections were treated with 3% H_2_O_2_ for 15 min at room temperature to inactivate peroxidase. Afterwards, they were rinsed three times with PBS for 2 min each to remove any residual H_2_O_2_. After washing with PBS, sections were pre-incubated in 0.4% Triton x-100 (T8200, Solarbio, Beijing, China) and 1% BSA (V900933, Sigma) and 4% goat serum (SL038, Solarbio) in PBS for 1 h at room temperature. The sections were then incubated with the primary antibody Iba1 (1:1000, 019-19741, FUJIFILM Wako Pure Chemical Corporation, Osaka, Japan), as well as NeuN (1:2000, ab177487, Abcam, Cambridge, UK) and GFAP (1:2000, ab7260, Abcam), overnight at 4 °C. On the following day, sections were washed in PBS and then exposed to horseradish peroxidase (HRP)-conjugated secondary antibody (1:2000, 31460, Invitrogen, Carlsbad, CA, USA) for 1 h at room temperature. Thereafter, sections were washed with PBS and incubated with DAB (ZLI-9019, ZSGB-BIO, Beijing, China). Images were captured under identical exposure conditions. Background subtraction was conducted with ImageJ software (version 1.53e) on the basis of non-immunoreactive areas. A uniform threshold was subsequently used to obtain the ratio of positively stained area to the total specified area. Quantitative analysis was performed in a blinded manner by experienced researchers.

### 2.11. Western Blot Analysis

Rats in each group were terminally anesthetized with 1% sodium pentobarbital (6 mL/kg); then, cardiac perfusion was conducted using PBS, finally retrieving the brain. The proteins were extracted by using RIPA (P0013B, Beyotime, Shanghai, China) mixed with 1% protease inhibitor (87785, Thermo Fisher Scientific, Waltham, MA, USA) and phosphatase inhibitor (K1015, APExBIO, Houston, TX, USA). Protein concentrations were measured using a BCA Kit (SW101-02, Seven, Beijing, China). Protein samples were separated by SDS-PAGE, and transferred to a PVDF membrane (IPVH00010, Millipore, Burlington, MA, USA). After blocking with 5% skimmed milk for 1 h, the membranes were probed with primary antibodies against MAP2 (1:1000, 17490-1-AP, Proteintech, Wuhai, China), NFL (1:500, 4747-MSM2-P1, Thermo Fisher Scientific), PSD95 (1:3000, ab238135, Abcam), L1CAM (1:1000, 20659-1-AP, Proteintech), MBP (1:1000, 10458-1-AP, Proteintech), and β-actin (1:2000, 66009-1-Ig, Proteintech) overnight at 4 °C. After washing three times with TBST, the membranes were probed with HRP-labeled secondary antibody (1:5000, ab7090, ab6789, Abcam) for 1 h. The binding signal was detected by ECL reagents (34580, Thermo Fisher Scientific). The images were collected by an imaging system (Bio-Rad, Hercules, CA, USA). The quantification of proteins was analyzed by the software Image J (version 1.53e), which measured the density of proteins.

### 2.12. Immunofluorescence Staining

After three washes with PBS, sections were blocked at room temperature for 1 h in PBS containing 0.3% Triton X-100 and 5% BSA. Subsequently, sections were incubated overnight at 4 °C with the following primary antibodies: anti-MAP2 (1:500; 17490-1-AP, Proteintech), anti-NFL (1:500; 4747-MSM2-P1, Thermo Fisher Scientific), and anti-PSD95 (1:1000; ab238135, Abcam). Then, sections were washed in PBS and then incubated for 1 h at room temperature in the dark with appropriate Alexa Fluor-conjugated secondary antibodies (1:2000; A-11012, Thermo Fisher Scientific). Nuclei were counterstained with DAPI (1:2000; C1002, Beyotime, China). Finally, sections were mounted using antifade mounting medium (BMU104, Abbkine, Wuhai, China) and imaged with a fluorescence microscope (CYTATION5, BioTek Instruments, Winooski, VT, USA).

### 2.13. Cell Culture and Kainic Acid Treatment

Primary hippocampal neurons were isolated from one-day-old Sprague Dawley rats. Following decapitation, the brains were quickly taken out, and the meninges and blood vessels were removed under a stereomicroscope. The hippocampi were isolated and enzymatically dissociated using papain (A501612-0025, Sangon Biotech, Shanghai, China) at 37 °C in a cell incubator for 20 min. Enzymatic digestion was terminated by adding culture medium containing 10% fetal bovine serum (16000-044, Gibco, Waltham, MA, USA), followed by gentle trituration with a 1 mL pipette in the presence of DNase I (D61780, Acmec Biochemical, Shanghai, China). After triturating 10 times, the cell suspension was passed through a 200-mesh filter and centrifuged to collect the supernatant. The cell pellet was resuspended and seeded onto poly-D-lysine-coated 6-well plates. After 4–6 h, the medium was replaced with neuron-specific culture medium composed of Neurobasal™ Plus (21103049, Gibco) supplemented with 2% B27 (A3582801, Gibco), 0.5 mM GlutaMAX (25030081, Gibco), and 1% penicillin/streptomycin (15140122, Gibco). Cultures were maintained at 37 °C in a humidified atmosphere of 5% CO_2_ in air. Neurons cultured for 10–14 days were used for subsequent experiments. HT22 cells which express L1CAM were cultured in Dulbecco’s modified Eagle’s medium (11965118, Gibco) supplemented with 10% fetal bovine serum (A5669701, Gibco) and 1% penicillin–streptomycin (15140122, Gibco) at 37 °C in a humidified atmosphere containing 5% CO_2_. For transfection, cells were seeded in 6-well plates and transfected with sL1 siRNA (5′-GCAAGAUCUUGCACAUCAATT-3′;5′-UUGAUGUGCAAGAUCUUGCTT-3′, Gene Pharma, Shanghai, China) using Lipofectamine 3000 (L3000150, Thermo Fisher Scientific) according to the manufacturer’s instructions.

For KA in vitro application, cultures of hippocampal neurons and HT22 cells were treated with 500 μM KA, and SVHRSP (50 μM) and anagrelide (20 μM) were added simultaneously with KA. After 48 h of treatment, cells were harvested for protein extraction.

### 2.14. Statistical Analysis

All data are presented as mean ± SEM. One-way analysis of variance was used for data analyses by GraphPad Prism 10.1.2 statistical software. *p* < 0.05 indicated that the differences were statistically significant.

## 3. Results

### 3.1. SVHRSP Reduces Spontaneous Seizure Activity in Epileptic Rats

**Figure 1:**
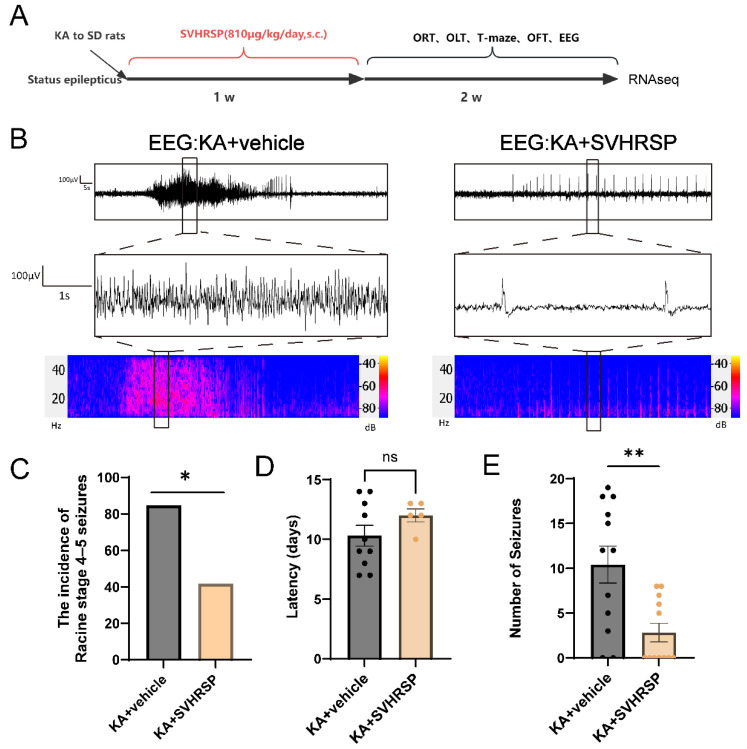
SVHRSP reduces spontaneous seizure activity in KA-induced epileptic rats. (**A**) A schematic diagram of the experimental design. (**B**) Representative 2 min EEG recordings during the seizure period (2 weeks after KA induction). (**C**) The overall incidence of stage 4 or higher seizures, analyzed by video monitoring. (**D**) Latency (days) in each group. (**E**) The number of spontaneous seizures in each group. *n* = 12 per group. ns, not significant; * *p* < 0.05, ** *p* < 0.01. Data are presented as mean  ±  SEM. Fisher’s exact test was used to determine seizure incidence (**C**), while the unpaired two-tailed Student’s *t*-test was used to analyze seizure latency (**D**) and seizure number (**E**).

We established a KA-induced TLE model in rats by the intraperitoneal injection of KA (10 mg/kg). Only animals that had reached Racine stages 4–5 within 1 h and exhibited status epilepticus lasting for at least 2 h were included and randomly assigned to undergo either vehicle or SVHRSP treatments for 7 consecutive days. This protocol helped minimize variability between groups and was used to assess the effects of SVHRSP on epileptogenesis (Figure 1A). Electroencephalogram (EEG) recordings from day 7 to day 14 post-KA administration revealed that compared with the KA group receiving only vehicle solution, SVHRSP-treated rats showed markedly shortened seizure duration, reduced seizure frequency, lower amplitude, fewer spike-wave discharges, and attenuated abnormal discharge energy (Figure 1B). Consistently, behavioral monitoring via video surveillance showed that the incidence of stage 4–5 seizures was lower in the SVHRSP-treated group compared to the KA group (Figure 1C). Furthermore, SVHRSP treatment prolonged the latency and decreased the number of spontaneous seizures in epileptic rats (Figure 1D). Collectively, these findings indicate that SVHRSP reduces the occurrence and progression of spontaneous seizures in the TLE model.

### 3.2. SVHRSP Alleviates Anxiety-like Behaviors and Cognitive Impairments in Epileptic Rats

**Figure 2:**
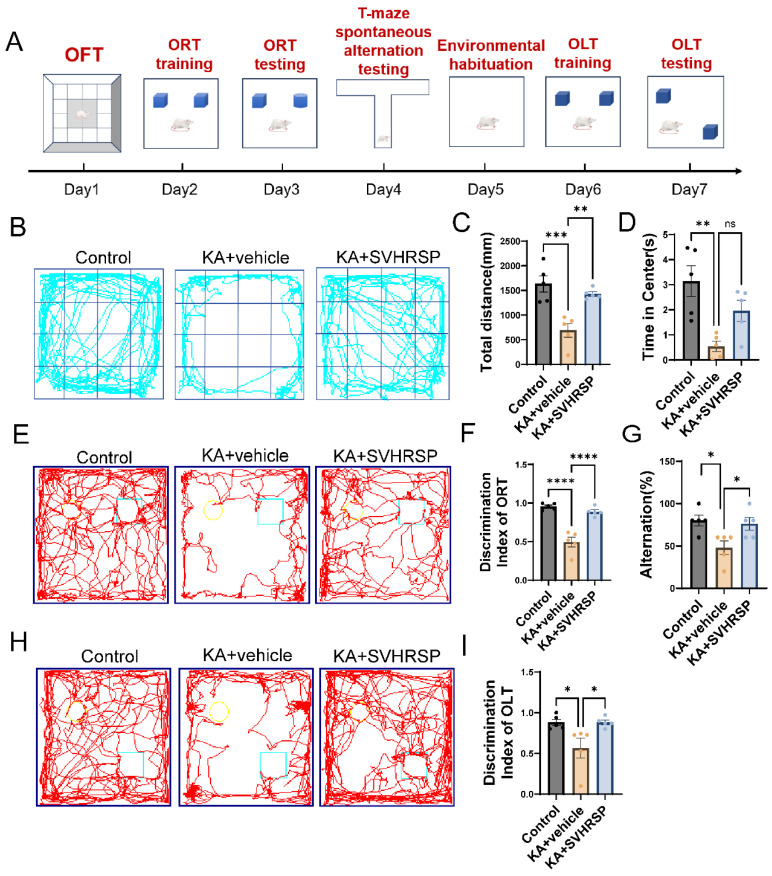
SVHRSP improves epilepsy-related cognitive deficits in rats. (**A**) A schematic diagram of the experimental design. (**B**) The track plots of rats in each group during the open-field test. (**C**) Total distance traveled by rats in the open-field test. (**D**) Time spent by rats in the central zone of the open-field test. (**E**) The track plots of rats in each group during the novel object recognition test (ORT). Circles and squares indicate the locations of the objects. (**F**) A statistical analysis of the discrimination index in the ORT. (**G**) A statistical analysis of the alternation scores (% correct choices) in the T-maze test. (**H**) The track plots of rats in each group during the novel object location test (OLT). Circles and squares indicate the locations of the objects. (**I**) A statistical analysis of the discrimination index in the OLT. *n* = 5 per group. ns, not significant; * *p* < 0.05, ** *p* < 0.01, *** *p* < 0.001, **** *p* < 0.0001, statistical significance was determined using one-way ANOVA followed by Tukey’s post hoc test. Data are presented as mean  ±  SEM.

Epilepsy is often associated with cognitive and behavioral deficits, including memory impairments, spatial memory dysfunction, anxiety-like behaviors, and executive dysfunction. To evaluate the effect of SVHRSP on epilepsy-related anxiety-like behaviors, we performed an open-field test, which facilitates a mild-stress test for determining anxiety levels in rodents. Rats in the KA group exhibited restricted movement predominantly along the periphery of the arena, avoidance of the central zone, reduced exploratory behavior toward the center, decreased total travel distance, and prolonged immobility time compared to control rats (Figure 2B). These observations indicated increased anxiety. Following SVHRSP treatment, rats showed an increase in the time spent in the central zone (Figure 2D) and a marked increase in total travel distance (Figure 2C), indicating that SVHRSP exerted an anxiolytic effect.

To further determine the effect of SVHRSP on epilepsy-related cognitive impairments, we assessed hippocampus-dependent short-term learning and memory performance using the novel object recognition test (ORT), the novel object location test (OLT), and the T-maze test. Compared to the control group, KA-treated rats exhibited spatial and non-spatial cognitive deficits, as evidenced by a marked reduction in the discrimination index in both ORT (Figure 2E,F) and OLT (Figure 2H,I), as well as decreased alternation scores in the T-maze test (Figure 2G). SVHRSP treatment improved the discrimination indices in ORT and OLT and increased the alternation scores in the T-maze test, indicating that SVHRSP ameliorated KA-induced deficits in short-term working memory.

### 3.3. SVHRSP Alleviates KA-Induced Hippocampal Sclerosis in Epileptic Rats

**Figure 3:**
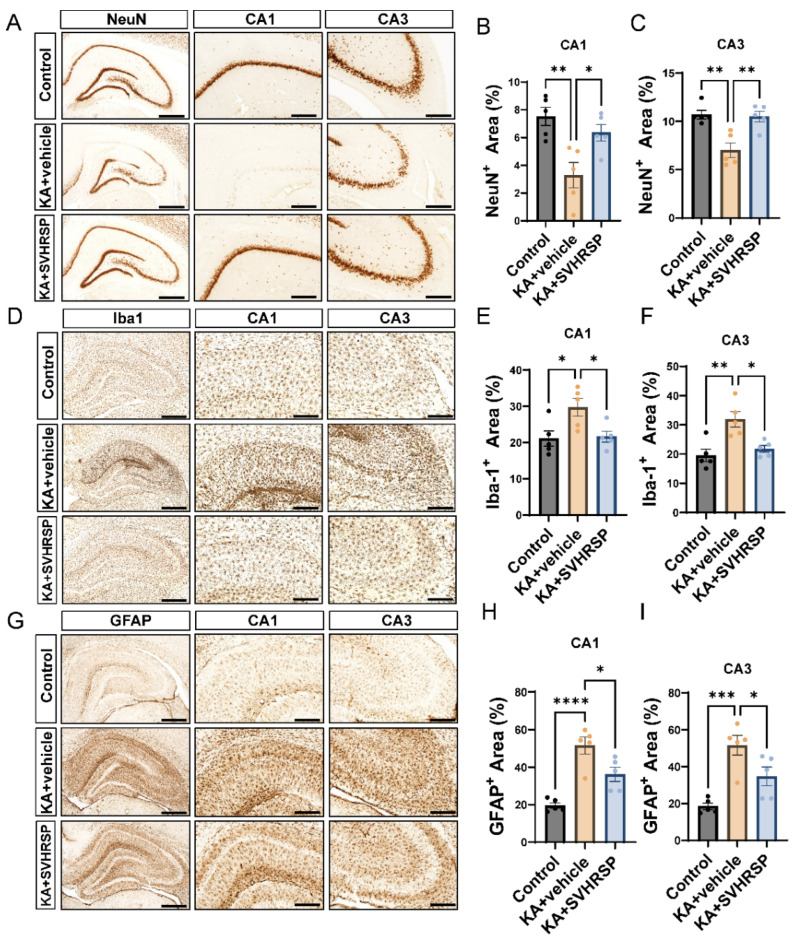
SVHRSP alleviates hippocampal sclerosis in epileptic rats. (**A**) Representative images of NeuN immunoreactivity in the hippocampus. (**B**) Quantification of the NeuN-positive area in CA1. (**C**) Quantification of the NeuN-positive area in CA3. (**D**) Representative images of Iba1 immunoreactivity. (**E**) Quantification of the Iba1-positive area in l CA1. (**F**) Quantification of the Iba1-positive area in CA3. (**G**) Representative images of GFAP immunoreactivity. (**H**) Quantification of the GFAP-positive area in CA1. (**I**) Quantification of the GFAP-positive area in CA3. *n* = 5 per group. Scale bars are 625 μm and 200 μm, respectively. * *p* < 0.05, ** *p* < 0.01, *** *p* < 0.001, **** *p* < 0.0001, statistical significance was determined using one-way ANOVA followed by Tukey’s post hoc test. Data are presented as mean  ±  SEM.

Hippocampal sclerosis is a major pathological feature of TLE, with seizures both contributing to and exacerbating this pathological condition. TLE is characterized by neuronal cell loss and gliosis. NeuN staining was used to quantify neurons. As shown in Figure 3A–C, A substantial loss of NeuN-positive neurons was observed in the hippocampal regions of epileptic rats, particularly in the CA1 and CA3 regions. SVHRSP treatment reduced the loss of pyramidal neurons induced by KA, demonstrating remarkable neuroprotective effects.

The activation of astrocytes and microglia was detected using the markers GFAP and Iba1. In epileptic rats, a large number of reactive microglia were observed in the hippocampal CA1 and CA3 regions, exhibiting enlarged cell bodies, shortened processes, and round or rod-shaped morphology. In contrast, the level of microglial activation in the SVHRSP-treated epileptic rats was reduced, with fewer Iba1-positive microglial cells showing activation (Figure 3D–F). Additionally, a large number of protoplasmic astrocytes were observed in the KA group, with increased GFAP staining and morphological changes (thicker processes) (Figure 3G–I). In the SVHRSP-treated group, the number of GFAP-positive cells was reduced. These results indicate that SVHRSP alleviates the pathological changes associated with hippocampal sclerosis in epileptic rats.

### 3.4. Transcriptomic Analysis Reveals That SVHRSP Enhances Neuroplasticity-Related Pathways Including Cell Adhesion Markers and Axon Guidance Cues

**Figure 4:**
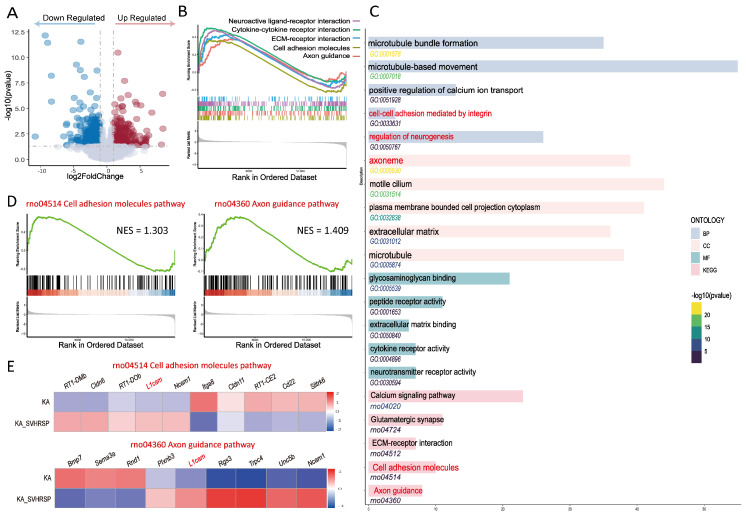
Transcriptomic analysis indicates that SVHRSP enhances neuroplasticity-related pathways, including cell adhesion and axon guidance. (**A**) A volcano plot showing DEGs in the hippocampus, with upregulated (red) and downregulated (blue) genes in the SVHRSP-treated group compared to the KA group (*p* < 0.05, |log_2_Fold Change| > 1). (**B**) The top five enriched signaling pathways identified by GSEA. (**C**) GO and KEGG enrichment analysis of DEGs. (**D**) GSEA results for axon guidance and cell adhesion pathways. (**E**) A heatmap showing the expression of genes associated with axon guidance and cell adhesion pathways.

To further investigate the molecular mechanisms underlying the anti-epileptic and neuroprotective effects of SVHRSP, we performed RNA sequencing on hippocampal tissue collected 2 weeks after KA application from the KA only control group and the KA + SVHRSP group. Compared to the KA group, a total of 897 differentially expressed genes were identified in the KA + SVHRSP group (*p* < 0.05), with 447 genes upregulated and 450 genes downregulated (Figure 4A). Gene Set Enrichment Analysis indicated that several pathways related to neuronal functions, including axon guidance, cell adhesion, extracellular matrix-receptor interaction, cytokine-cytokine receptor interaction, and neuroactive ligand–receptor interaction, were enriched (Figure 4B). Further analysis of Gene Ontology and Kyoto Encyclopedia of Genes and Genomes pathways confirmed that axon guidance and cell adhesion molecule-triggered pathways were consistently enriched across Gene Set Enrichment Analysis GSEA, Kyoto Encyclopedia of Genes and Genomes KEGG, and Gene Ontology GO analyses (Figure 4C). These findings suggested that SVHRSP may exert its anti-epileptogenic and neuroprotective effects through modulation of cell adhesion molecule-triggered pathways, including axon guidance. To explore this, we performed a cluster analysis of genes in these pathways. Heatmap analysis revealed that neural adhesion molecules, including *L1cam* and *Ncam*, were upregulated in the hippocampus of SVHRSP-treated rats (Figure 4E). Additionally, GSEA focusing on individual pathways showed that, compared with the epileptic group, epileptic rats in the SVHRSP-treated group exhibited an upregulation of genes involved in axon guidance (rno04514, NES = 1.303) and cell adhesion (rno04360, NES = 1.409) (Figure 4D). Collectively, these results indicate that SVHRSP restores neuroplasticity-associated signaling pathways, particularly those governing cell adhesion and axon guidance, suggesting that they may contribute to the peptide’s therapeutic effects in epilepsy.

### 3.5. SVHRSP Attenuates Abnormalities of Neuronal Markers in Epileptic Rats

**Figure 5:**
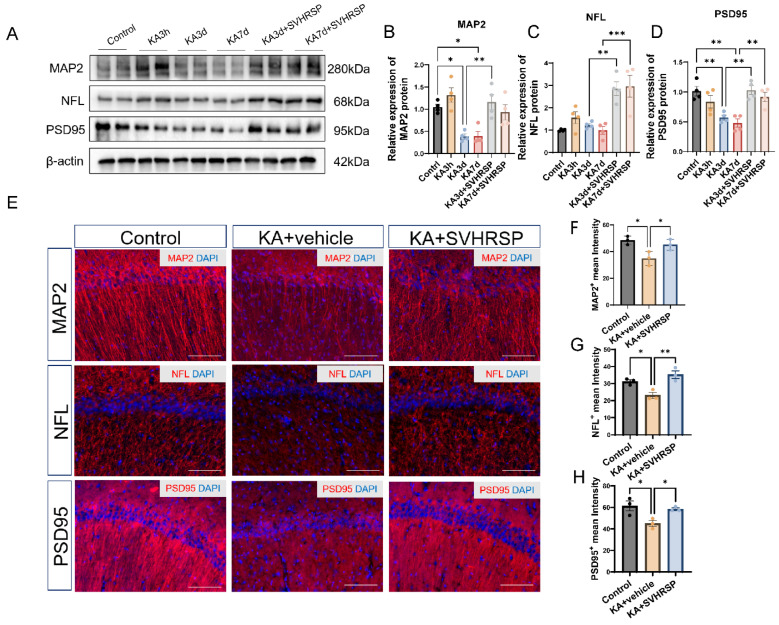
SVHRSP attenuates neuronal damage in epileptic rats. (**A**) Representative Western blot images of MAP2, NFL, and PSD95 expression at 3 h, 3 days, and 7 days after KA treatment, and followed by SVHRSP treatment for 3 and 7 days. (**B**) Quantification of MAP2 protein expression. (**C**) Quantification of NFL protein expression. (**D**) Quantification of PSD95 protein expression. (**E**) Representative images of MAP2, NFL, PSD95 immunoreactivities in the hippocampus 7 days after KA application. Scale bar, 200 μm. (**F**–**H**) Quantification of average optical density for MAP2, NFL, and PSD95. Statistical significance was determined using one-way ANOVA followed by Tukey’s post hoc test. Data are presented as mean  ±  SEM, * *p* < 0.05, ** *p* < 0.01, *** *p* < 0.001, *n* = 3–4 per group.

Dendrites, axons, and synapses are critical structures for information transmission between neurons. Damage to these structures disrupts normal neural signaling, leading to abnormal discharges and cognitive and behavioral dysfunctions. Microtubule-associated protein 2 (MAP2) is a key structural protein of the neuronal cytoskeleton, primarily localized in dendrites, where it regulates cytoskeletal organization and function through tight binding to microtubules. Neurofilament light chain (NFL) is a major intermediate filament protein of the neuronal cytoskeleton, predominantly expressed in axons. Postsynaptic density protein 95 (PSD95) is a scaffolding protein located in the postsynaptic density that regulates synaptic plasticity and neurotransmission efficiency, playing essential roles in maintaining synaptic structure and function. Our study showed that at 3 h after KA treatment, the levels of MAP2 and NFL were increased compared to controls. However, at 3 and 7 days after KA application, MAP2 expression was reduced. SVHRSP treatment restored MAP2 expression at both 3 and 7 days post-SE. Moreover, SVHRSP treatment also increased the expression of NFL and PSD95 (Figure 5A–D). Immunofluorescence staining performed on hippocampal sections 7 days after KA insult further confirmed these findings. Compared with the control group, the KA group exhibited reduced expression levels of neurite-associated proteins. SVHRSP treatment reversed these alterations (Figure 5E–H).

### 3.6. SVHRSP Increases the Expression of a L1CAM Fragment in Epileptic Rats

**Figure 6:**
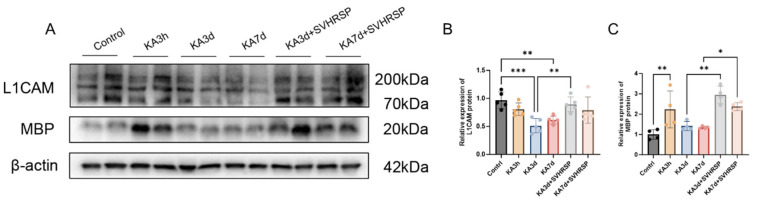
SVHRSP increases the expression of a L1CAM fragment in epileptic rats. (**A**) Representative Western blot images of L1CAM and MBP expression at 3 h, 3 days, and 7 days after KA administration followed by SVHRSP treatment for 3 and 7 days. (**B**) Quantification of L1CAM protein levels. (**C**) Quantification of MBP levels. Statistical significance was determined using one-way ANOVA followed by Tukey’s post hoc test. Data are presented as mean  ±  SEM. * *p* < 0.05, ** *p* < 0.01, *** *p* < 0.001, *n* = 4–5 per group.

Myelin basic protein (MBP) has been identified as a serine protease that cleaves L1CAM at the Arg687 site within its extracellular domain, generating a 70 kDa transmembrane fragment (L1-70) that promotes neurite outgrowth and neuronal survival in cultured cells [13]. Three hours after kainic acid application (KA 3h), MBP expression was elevated compared to the control group. By 3 and 7 days after KA application, MBP expression returned to levels comparable to controls (Figure 6A,C). Notably, SVHRSP treatment for 3 and 7 days increased MBP expression and enhanced levels of the L1-70 fragment (Figure 6A,B).

### 3.7. L1CAM Mediates the Protective Effects of SVHRSP Against Damage of Cultured Neurons

**Figure 7:**
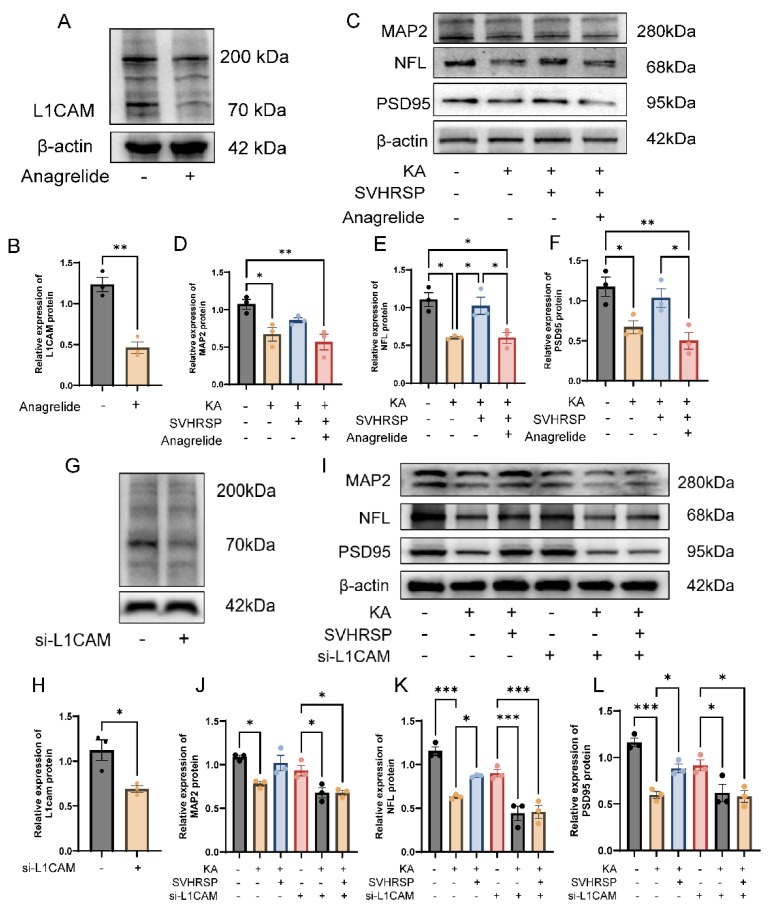
L1CAM contributes to the protective effects of SVHRSP against damage in cultured neurons. (**A**–**F**) Representative Western blot images and quantification of L1CAM, MAP2, NFL, and PSD95 in cultures of hippocampal neurons. Western blots of (**A**) L1CAM; (**B**) MAP2, NFL, and PSD95; (**C**–**F**) Quantification of L1CAM, MAP2, NFL, and PSD95. (**G**–**L**) Representative Western blot images and quantification of L1CAM, MAP2, NFL, and PSD95 in HT22 cells. Western blots of (**G**) L1CAM; (**H**) MAP2, NFL, and PSD95; (**I**–**L**) Quantification of L1CAM, MAP2, NFL, and PSD95. Statistical significance was determined using one-way ANOVA followed by Tukey’s post hoc test. Data are presented mean  ±  SEM, * *p* < 0.05, ** *p* < 0.01, *** *p* < 0.001, *n* = 3 per group.

Anagrelide, commonly used to treat thrombocythemia by preventing megakaryocyte maturation and reducing platelet counts, has been identified as an antagonistic L1 mimetic [30]. In cultured neurons, anagrelide inhibited the expression of L1CAM protein (Figure 7A,B). Compared to the control group, KA treatment markedly downregulated the expression of dendritic, axonal, and synaptic markers. SVHRSP treatment reversed these reductions (Figure 7C–F). To address the question regarding potential off-target effects of the antagonistic L1CAM mimetic anagrelide, we applied L1CAM-specific siRNA to cultured L1 immunoreactive HT22 cells (Figure 7G–L). Notably, both the functional inhibition and genetic knockdown of L1CAM attenuated the protective effects of SVHRSP against KA-induced neuronal damage.

## 4. Discussion

In this study, we demonstrated for the first time that the SVHRSP scorpion venom peptide from *Buthus martensii Karsch* scorpion remarkably suppressed spontaneous seizures, improved cognitive impairments, and alleviated anxiety-like behaviors in KA-induced epileptic rats. SVHRSP treatment also alleviated hippocampal sclerosis, preserved neurite integrity, and increased the expression of markers for dendrites, axons, and synapses. In addition, transcriptomic analysis revealed that SVHRSP specifically upregulated genes involved in adhesion molecule-triggered pathways and axon guidance. Of note, SVHRSP treatment also enhanced L1CAM expression, suggesting that SVHRSP may prevent synaptic loss, seizure development, and cognitive deficits by regulating the expression of the multifunctional L1CAM. Furthermore, the neuroprotective effects of SVHRSP were attenuated by the L1 antagonistic mimetic anagrelide L1CAM in primary hippocampal neurons and by L1CAM knockdown via siRNA in HT22 cells, supporting the notion that L1CAM mediates SVHRSP’s neuroprotective actions.

Hippocampal sclerosis, characterized by selective neuronal loss and gliosis [31], is a hallmark of the pathological features of clinical TLE. These histopathological changes not only disrupt hippocampal architecture but also lead to aberrant neural network remodeling, which underlies the persistence of seizures and cognitive decline. In the present study, SVHRSP treatment attenuated KA-induced hippocampal sclerosis and ameliorated the associated dendritic, axonal, and synaptic degeneration. Notably, transcriptomic analysis revealed that SVHRSP selectively upregulated genes associated with cell adhesion molecule-triggered pathways and axon guidance, suggesting that the modulation of neural plasticity–related pathways may underlie the peptide’s ability to suppress epileptic network remodeling. This finding is consistent with previous single-cell transcriptomic studies showing sublayer- and cell-type-specific transcriptional alterations in the CA1 region during hippocampal sclerosis, particularly affecting genes related to synaptic function and structure, including those encoding adhesion and axon guidance molecules [6].

Axon guidance molecules, such as the immunoglobulin, netrin, and semaphorin families, have been implicated in the formation of aberrant synaptic connections in epilepsy [32]. During nervous system development, axon guidance is critical for establishing functional circuits by directing axons to their targets [33]. After birth, axon guidance molecules continue to be expressed at excitatory and inhibitory synapses, contributing to the circuit refinement and maintenance of the excitation–inhibition balance. For example, recombinant Sema3F increases excitatory synaptic transmission in cerebellar granule cells and CA1 pyramidal neurons [34]. Evidence suggests that disruptions in axon guidance are closely linked to epilepsy and related neuropathological processes such as inflammation, synaptic plasticity alterations, and mossy fiber sprouting [35]. The Sema3F/NTP-2 family, for instance, modulates mossy fiber sprouting and affects seizure development [36], highlighting its important contribution to axon guidance in shaping neuronal connectivity and signal transmission in epilepsy. In addition, the loss or dysfunction of cell adhesion molecules has been shown to increase seizure susceptibility and compromise neural network stability [37]. Disruptions in network assembly may weaken endogenous anti-epileptogenic mechanisms, impair drug efficacy, and contribute to the development of drug-resistant epilepsy [38,39].

In these functional roles, cell adhesion molecules are essential for synapse functions. Through trans-synaptic signaling, cell adhesion molecules impinge on all aspects of neural network activities. Several studies have linked their dysfunction to different types of neurological diseases, including epilepsy. In epilepsy, abnormal adhesion molecule function disrupts synaptic connectivity, alters neuronal excitability, and affects network activity [37]. For example, interactions between neuroligins and neurexins maintain the balance between excitatory and inhibitory synapses. Increased expression of neuroligin-1 and its binding partner neurexin-1β has been observed in epileptic foci, and the knockdown of neuroligin-1 alleviates seizure frequency and severity in epileptic rats [40]. Also, the abnormal expression of N-cadherin, like for L1CAM, has been associated with hippocampal synaptic remodeling and mossy fiber sprouting [41], which are key features in the pathogenesis of epilepsy. Eph receptors and Ephrin ligands also contribute to synaptic plasticity and epileptogenesis, with EphB receptor expression being markedly upregulated in epileptic patients and animal models of epilepsy [42].

L1CAM was the first adhesion molecule to be studied extensively in nervous system functions. As a member of the immunoglobulin superfamily, it plays a pivotal role in all aspects of neural development, plasticity, and regeneration [43]. L1CAM is an autophagic receptor that reduces the accumulation of aberrant proteins, such as Abeta [44]. A transmembrane fragment of L1CAM enters mitochondria and increases their activity [45]. The intracellular domain of L1CAM regulates transcription of genes [46]. It interacts with intracellular cytoskeletal elements, including microtubule-associated proteins and ankyrins. It cooperates with extracellular matrix components such as chondroitin sulfate and hyaluronic acid to maintain long-term synaptic homeostasis [11]. In the adult brain, L1CAM remains expressed and regulates excitatory and inhibitory synaptic plasticity by mediating synaptic pruning and stabilization. Specifically, members of the L1 family, including NrCAM and CHL1, interact with Neuropilin and Plexin receptor complexes to mediate Semaphorin signaling, thereby promoting dendritic spine pruning and synaptic remodeling [47,48]. Synaptic pruning abnormalities are implicated in several neurodevelopmental disorders, including autism spectrum disorder and schizophrenia [49,50]. Since no studies on L1CAM in epilepsy have been carried out, we expected that its dysfunction would disrupt the excitatory–inhibitory synaptic balance and thereby increase seizure susceptibility [51]. Its dysfunction could impair network dynamics, contributing to abnormal synchronization during seizures [52].

Epileptogenesis is a multifactorial process involving complex interference between neuronal injury, glial activation, neuroinflammation, oxidative stress, and aberrant synaptic remodeling. Among these, oxidative stress and inflammation are widely recognized as early and persistent contributors to the development of epileptic network dysfunction [53,54,55], leading to neuronal loss, gliosis, and an imbalance between excitatory and inhibitory signaling. In the present study, SVHRSP treatment alleviated the severity of spontaneous seizures and reduced glial cell proliferation, while increasing the expression of neuronal markers. Transcriptomic analysis revealed that SVHRSP increased L1CAM expression. To determine whether this effect is mediated by L1CAM, we first performed experiments in cultured neurons and found that the L1CAM inhibitor anagrelide reduced the neuroprotective effects of SVHRSP. Given the potential off-target effects of anagrelide, we used HT22 cells, which express L1CAM, and applied L1CAM-specific siRNA. Silencing L1CAM similarly attenuated the ability of SVHRSP to upregulate neuronal markers, supporting a role for L1CAM in mediating its neuroprotective effects. However, the causal relationship between L1CAM modulation and seizure suppression remains to be elucidated. This is important to consider, since other molecules than L1CAM are likely to contribute to the neuroprotective effects of SVHRSP. Recent findings have highlighted the potential regulatory role of L1CAM in neuroinflammation and oxidative stress [56]. Specifically, L1CAM has been shown to attenuate microglial activation and nitric oxide production under inflammatory conditions, thereby promoting an anti-inflammatory function and preserving neuronal viability. Taken together, L1CAM may represent a promising target in epilepsy research, offering potential insights into the underlying mechanisms of synaptic functions, neuroimmune regulation, and neural circuit remodeling.

Our study has several limitations. First, though our findings reached statistical significance, the sample sizes for both behavioral and molecular analyses were relatively small and limit the broader generalizability of the results. Second, the use of an 80 × 80 cm open-field arena, which is a little smaller than the ideal size for adult rats (≥100 × 100 cm). The limited space may have restricted locomotor exploration and inflated measurements of time in the center. Third, although we spaced out the behavioral tasks and thoroughly cleaned and disinfected the arena between sessions, the repeated use of the same arena across multiple tasks may have introduced familiarity effects. Fourth, while our findings suggest that SVHRSP confers neuroprotection at least in part through L1CAM, the causal relationship between L1CAM and seizure suppression remains to be clarified in detail. Further studies using cell type-specific genetic manipulations and in vivo imaging approaches will help to elucidate these pathways and to confirm causality.

Altogether, in the present study, we obtained first evidence that L1CAM maintains neuronal integrity in both KA-induced epileptic rats and in vitro neuronal models. However, transcriptomic analysis following SVHRSP treatment revealed broader regulatory effects, including enrichment in pathways related to calcium signaling, axon guidance and extracellular matrix interactions. While not directly indicated, indirect effects on neuroinflammation, ion channel regulation, or synaptic remodeling may also contribute to the protective role of SVHRSP. Future studies incorporating other adhesion molecules, analyses of kinetics, loss-of-function models, and pathway-specific interventions are needed to clarify these relationships and elucidate the mechanistic basis of SVHRSP’s anti-epileptic actions.

## 5. Conclusions

Our findings indicate that SVHRSP alleviates spontaneous seizures in KA-induced epileptic rats, improves cognitive and emotional behaviors, and mitigates hippocampal neuronal damage and glial activation. SVHRSP exerts its neuroprotective effects by enhancing the expression of the cell adhesion molecule L1 and its functional fragment L1-70. These results provide valuable insights into the potential of SVHRSP as a therapeutic agent for drug-resistant epilepsy.

## Data Availability

The original contributions presented in this study are included in the Supplementary Materials; further inquiries can be directed to the corresponding authors.

## Supplementary Materials

The following supporting information can be downloaded at: https://www.mdpi.com/article/10.3390/biom15071032/s1, Figure S1: Epilepsy-inducing parameters Racine score, initial latenc; Figure S2: WuXi AppTec’s results in a study evaluating the pharmacodynamics of SVHRSP on a model of KA-induced chronic epilepsy in rat; Figure S3: Original blots for Figure 7A,C,G,I showing in vitro experiments with siRNA-mediated knockdown of L1CAM in HT22 cells (*n* = 3); Figure S4: Original blots for Figure 6A showing the expression of L1CAM and MBP in the rat hippocampus (*n* = 4); Table S1: Epilepsy-inducing parameters.

## Abbreviations

AED	Anti-epileptic Drug
ANOVA	Analysis of Variance
BSA	Bovine Serum Albumin
CAMs	Cell Adhesion Molecules
DAPI	4′,6-Diamidino-2-Phenylindole
DBP	Disulfide-Bridged Peptides
DEG	Differentially Expressed Gene
DI	Discrimination Index
EEG	Electroencephalogram
FPKM	Fragments Per Kilobase of Transcript Per Million Mapped Reads
GFAP	Glial Fibrillary Acidic Protein
GO	Gene Ontology
GSEA	Gene Set Enrichment Analysis
HRP	Horseradish Peroxidase
KA	Kainic Acid
KEGG	Kyoto Encyclopedia of Genes and Genomes
L1-70	70 kDa Functional Fragment of L1CAM
L1CAM	L1 Cell Adhesion Molecule
MAP2	Microtubule-Associated Protein 2
MBP	Myelin Basic Protein
NDBP	Non-Disulfide-Bridged Peptides
NFL	Neurofilament Light Chain
OLT	Object Location Test
OFT	Open-Field Test
ORT	Object Recognition Test
PBS	Phosphate-Buffered Saline
PSD95	Postsynaptic Density Protein 95
qPCR	Quantitative Polymerase Chain Reaction
SDS-PAGE	Sodium Dodecyl Sulfate Polyacrylamide Gel Electrophoresis
SE	Status Epilepticus
SEM	Standard Error of the Mean
SVHRSP	Scorpion Venom Heat-Resistant Synthetic Peptide
SRS	Spontaneous Recurrent Seizures
TBST	Tris-Buffered Saline with Tween 20
TLE	Temporal Lobe Epilepsy
