# Supplementary material for: The Upregulation of L1CAM by SVHRSP Mitigates Neuron Damage, Spontaneous Seizures, and Cognitive Dysfunction in a Kainic Acid-Induced Rat Model of Epilepsy
Source: Biomolecules. 2025 Jul 17;15(7):1032. doi: 10.3390/biom15071032 (PMC12293612; doi:10.3390/biom15071032)
Supplement: Supplementary file 1 [file biomolecules-15-01032-s001.zip › biomolecules-3652462-supplementary.pdf]

Supplementary Materials for

The Upregulation of L1CAM by SVHRSP Mitigates Neuron Damage, Spontaneous Seizures, and Cognitive Dysfunction in a Kainic Acid-Induced Rat Model of Epilepsy

This PDF file includes:

Figures S1–S4

Table S1

Supplementary Materials:

Table S1. Epilepsy-inducing parameters

Animal number	latency period (min)	Racine stage	SE Duration
E57	34	4	≥2h
E58	48	5	≥2h
E59	47	4	≥2h
E60	18	5	≥2h
E61	35	5	≥2h
E62	35	5	≥2h
E63	26	5	≥2h
E64	60	5	≥2h
E65	26	5	≥2h
E66	38	4	≥2h
E67	45	4	≥2h
E68	27	5	≥2h
E69	33	4	≥2h
E70	50	5	≥2h
E71	41	4	≥2h
E72	29	5	≥2h
E73	47	5	≥2h
E74	38	5	≥2h

E75	26	4	≥2h
E76	44	5	≥2h
E77	39	5	≥2h
E78	31	4	≥2h
E79	50	5	≥2h
E80	25	4	≥2h
E81	46	5	≥2h
E82	35	4	≥2h
E83	38	5	≥2h

Rats numbered E63, E71, and E81 died during subsequent experiments and were highlighted in yellow.

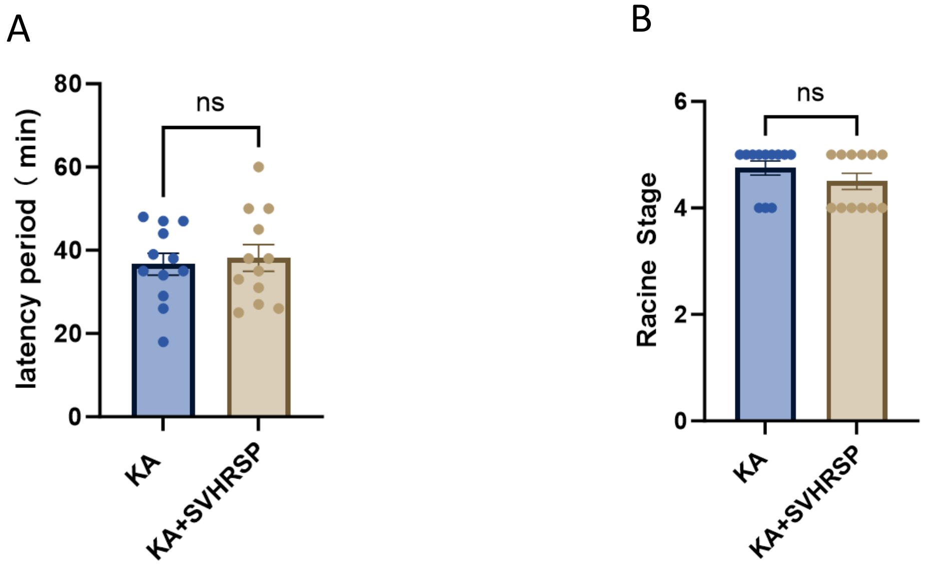

Figure S1. Epilepsy-inducing parameters Racine score, initial latency. (A) The latency period of status epilepticus in each group after KA injection. **(B)** The racine stage of each group after KA injection. n = 12. ns = $p < 0.05$. Data are presented as mean ± SEM. Statistical significance was determined using an unpaired two-tailed Student's *t*-test.

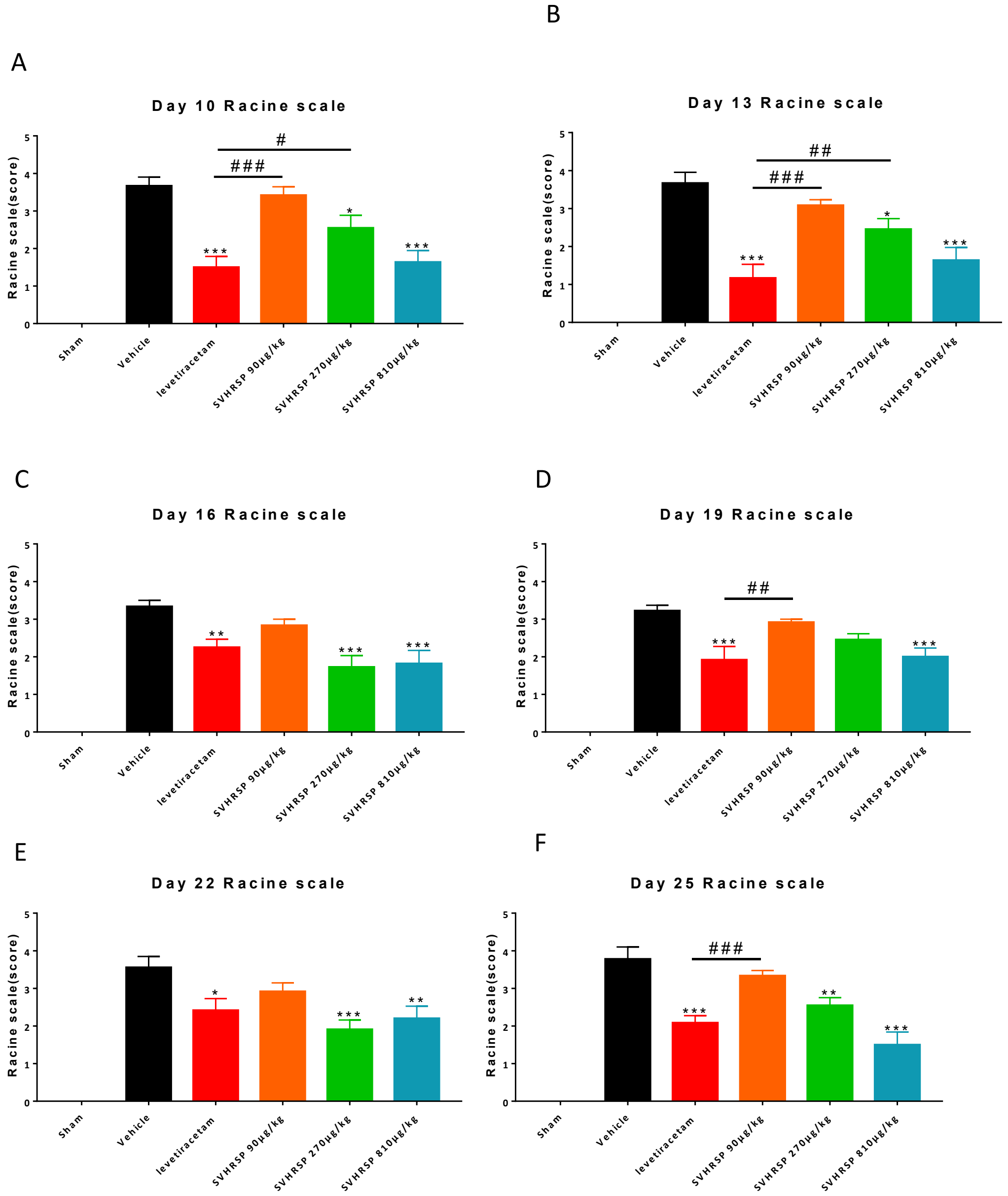

Figure S2. WuXi AppTec's results in a study evaluating the pharmacodynamics of SVHRSP on a model of KA-induced chronic epilepsy in rats. (A–F) Epilepsy Racine score of rats in different groups at different days. * $p < 0.05$, ** $p < 0.01$, *** $p < 0.001$, compared with the Vehicle group, $^{\#\#}$ $p < 0.01$, $^{\#\#\#}$ $p < 0.001$, compared with the positive control group. Data are presented as mean ± SEM. Statistical significance was determined using One-way ANOVA, Dunnett post hoc, Vehicle group N = 9, SVHRSP medium-dose group N = 11, SVHRSP high-dose group N = 11, and the other groups N = 12.

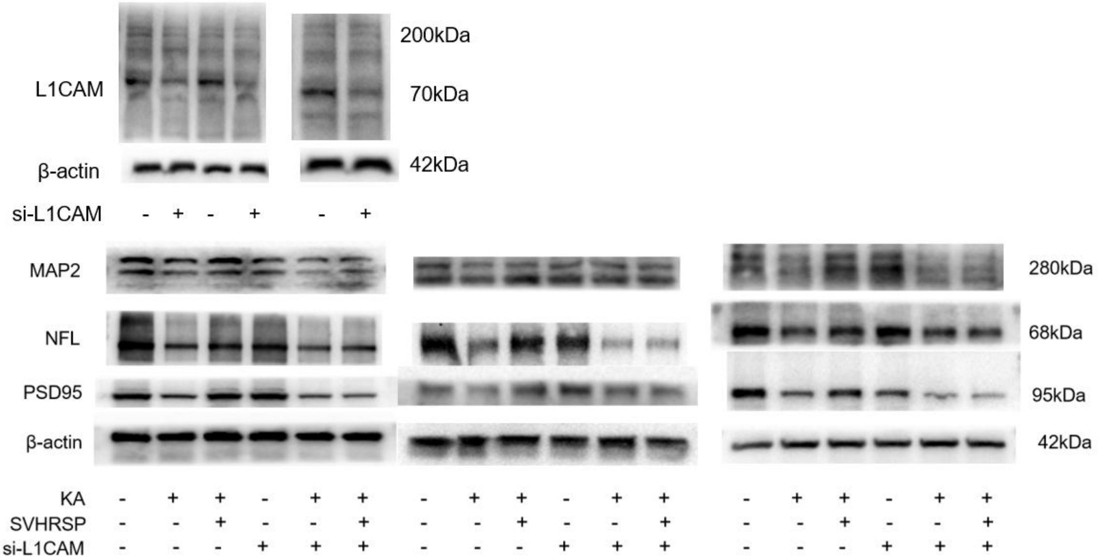

Figure S3. Original blots for Figure 7AC,G,I showing in vitro experiments with siRNA-mediated knockdown of L1CAM in HT22 cells (n = 3).

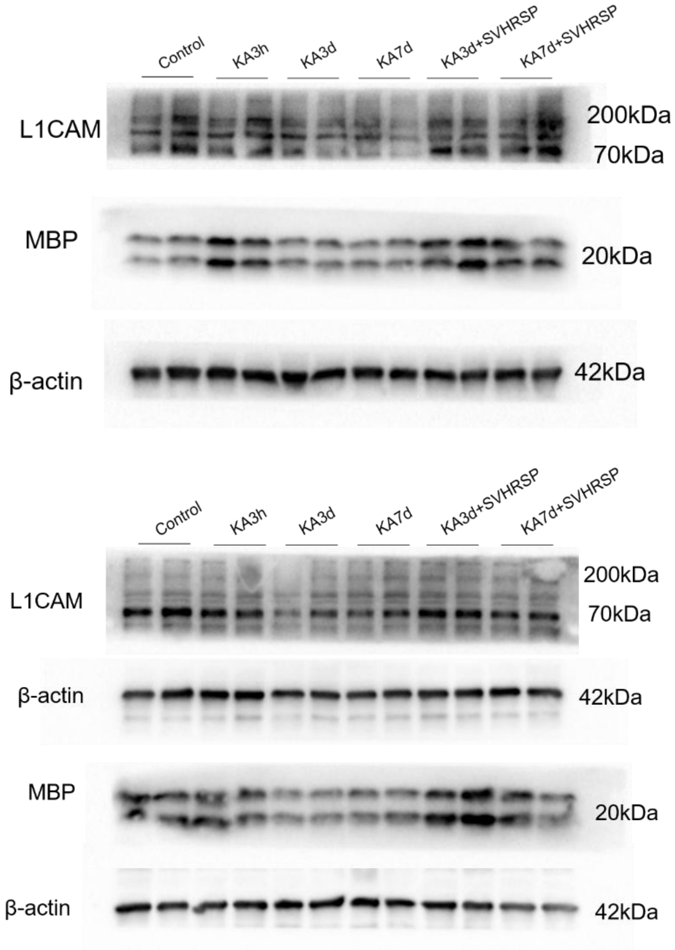

Figure S4. Original blots for Figure 6A showing the expression of L1CAM and MBP in the hippocampus (n = 4).
